# 2-*tert*-Butyl-1-(4-nitro­amino-1,2,5-oxadiazol-3-yl)diazene 1-oxide

**DOI:** 10.1107/S1600536812027353

**Published:** 2012-06-23

**Authors:** Xiang-Zhi Li, Bo-Zhou Wang, Xue-Zhong Fan, Seik Weng Ng

**Affiliations:** a Xi’an Modern Chemistry Research Institute, Xi’an 710065, People’s Republic of China; b Department of Chemistry, University of Malaya, 50603 Kuala Lumpur, Malaysia; c Chemistry Department, King Abdulaziz University, PO Box 80203 Jeddah, Saudi Arabia

## Abstract

In the title compound, C_6_H_10_N_6_O_4_, the nitro­amine –NHNO_2_ substituent and the C–N=N(→ O) unit of the other substituent of the oxadiazole ring are nearly coplanar with the five-membered ring [dihedral angles = 5.7 (1) and 3.0 (1)°]. The amino group of the –NHNO_2_ substituent is a hydrogen-bond donor to the two-coordinate N atom of the C—N=N(→ O) unit.

## Related literature

The synthesis required several steps; see: Churakov *et al.* (1995 ▶); Li *et al.* (2008 ▶); Mel’nikova *et al.* (2001 ▶). 
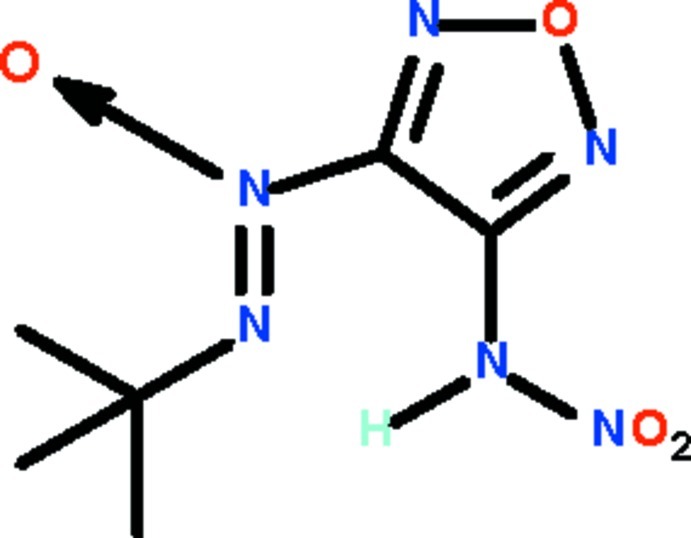


## Experimental

### 

#### Crystal data

C_6_H_10_N_6_O_4_
*M* *_r_* = 230.20
Monoclinic, 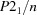

*a* = 6.2509 (5) Å
*b* = 9.1327 (8) Å
*c* = 18.6566 (16) Å
β = 92.134 (2)°
*V* = 1064.32 (16) Å^3^
*Z* = 4
Mo *K*α radiation
μ = 0.12 mm^−1^
*T* = 293 K
0.32 × 0.22 × 0.18 mm

#### Data collection

Bruker SMART APEX diffractometer
6176 measured reflections
2402 independent reflections
1711 reflections with *I* > 2σ(*I*)
*R*_int_ = 0.023

#### Refinement

*R*[*F* ^2^ > 2σ(*F* ^2^)] = 0.050
*wR*(*F* ^2^) = 0.166
*S* = 1.05
2402 reflections
149 parameters
1 restraint
H atoms treated by a mixture of independent and constrained refinement
Δρ_max_ = 0.21 e Å^−3^
Δρ_min_ = −0.21 e Å^−3^

### 

Data collection: *APEX2* (Bruker, 2007 ▶); cell refinement: *SAINT* (Bruker, 2007 ▶); data reduction: *SAINT*; program(s) used to solve structure: *SHELXS97* (Sheldrick, 2008 ▶); program(s) used to refine structure: *SHELXL97* (Sheldrick, 2008 ▶); molecular graphics: *X-SEED* (Barbour, 2001 ▶); software used to prepare material for publication: *publCIF* (Westrip, 2010 ▶).

## Supplementary Material

Crystal structure: contains datablock(s) global, I. DOI: 10.1107/S1600536812027353/xu5568sup1.cif

Structure factors: contains datablock(s) I. DOI: 10.1107/S1600536812027353/xu5568Isup2.hkl

Supplementary material file. DOI: 10.1107/S1600536812027353/xu5568Isup3.cml

Additional supplementary materials:  crystallographic information; 3D view; checkCIF report

## Figures and Tables

**Table 1:** Hydrogen-bond geometry (Å, °)

*D*—H⋯*A*	*D*—H	H⋯*A*	*D*⋯*A*	*D*—H⋯*A*
N5—H1⋯N1	0.87 (1)	2.18 (2)	2.758 (2)	124 (2)

**Fig. 1.**
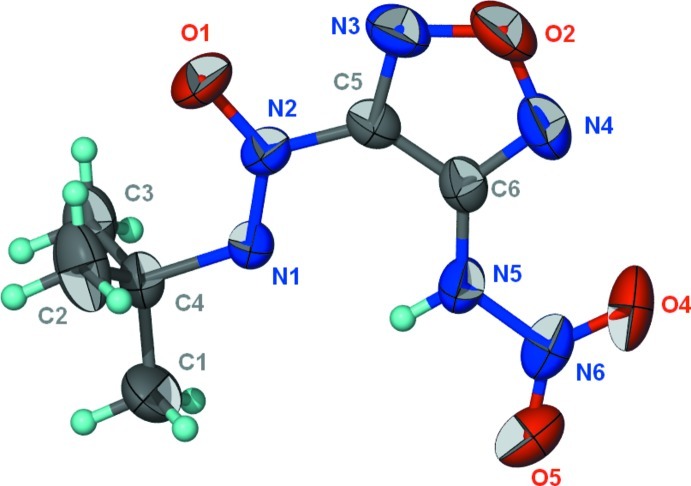
Thermal ellipsoid plot (Barbour, 2001) of C6H10N6O4 at the 50% probability level; hydrogen atoms are drawn as spheres of arbitrary radius.

## supplementary crystallographic information

### Comment

The title compound (Scheme I) as well as the precusor compounds (Churakov *et al.*, 1995; Li *et al.*, 2008; Mel'nikova *et al.*, 2001) represent a class of high energy materials that has a low hydrogen content in the molecular formula. The nitroamine –NHNO_2_ substituent and the C–N═ N(→O) unit of the second substituent of the oxadiazole ring of C_6_H_10_N_6_O_4_ are nearly coplanar with the five-membered ring [dihedral angles 5.7 (1), 3.0 (1) °] (Fig. 1). The amino group of the –NHNO_2_ substituent is hydrogen bond donor to the two-coordinate N atom of C–N═ N(→O) (Table 1).

### Experimental

The steps given below were adapted from published procedures (Churakov *et al.*, 1995; Li *et al.*, 2008; Mel'nikova *et al.*, 2001)..

Synthesis of 3-amino-4-nitrosofurazan

To a mixture of benzene (200 ml), 30% hydrogen peroxide (145 ml, 1.29 mol) and sodium tungstate dihydrate (16.5 g, 0.05 mol), concentrated sulfuric acid (10 ml, 180 mmol) was added dropwise at 278–283 K followed by diaminofurazan (10.8 g, 0.10 mmol). The mixture was kept stirred at 288 K for 1.5 h. The organic layer was separated, washed with water and dried over magnesium sulfate. The solvent was removed to yield a yellow solid (5.19 g, 90% yield). CH&N elemental analysis. Calc. for C_2_H_2_N_4_O_2_: C 21.06, H 1.77, N 49.12%. Found: C 20.82, H 1.77, N 48.95%.

Synthesis of 3-amino-4-*(tert*-butyl-NNO-azoxy)furazan

A suspension solution of *N*,*N*-dibromo-*tert*-butylamine (11.5 g, 50 mmol), cuprous chloride (10 g, 0.1 mol), and the above compound (50 mmol) in dichloromethane (500 ml) was stirred at 288–298 K for 15 h. The reaction mixture was poured into ice-water (500 ml). Sodium thiosulfate was then added. The organic layer was separated, washed with water and dried over magnesium sulfate. The solvent was removed to give a yellow solid (6.8 g, 70%). CH&N elemental analysis. Calc. for C_6_H_11_N_5_O_2_: C 38.92, H 5.99, N37.82%. Found: C 39.36, H 5.96, N 35.60%.

Synthesis of 3-nitramino-4-(*tert*-butyl-NNO-azoxy)furazan

To a stirred and cooled (273 K) solution of the above compound (2 g, 10.8 mmol) in carbon tetrachloride, concentrated nitric acid (2.72 g, 21.6 mmol) was added. Thesolution was stirred for 2 h after after which the temperature was raised to room temperature. The solvent was removed. Dichloromethane (100 ml) was added and the organic phase was washed with cold water (20 ml). The aqueous layer was extracted with more dichloromethyane (2×50 ml). The combined organic layer was dried over magnesium sulfate, filtered and the solvent was removed to give the title compound as a yellow solid (2.47 g, 100% yield). CH&N elemental analysis. Calc. for C_6_H_1_N_6_O_3_: C31.30, H 4.35, N 36.52%. Found: C 31.73, H 4.41, N 36.12%. Crystals were obtained upon recrystallization from dichloromethane.

### Refinement

Carbon-bound H-atoms were placed in calculated positions (C–H 0.96 Å) and were included in the refinement in the riding model approximation, with *U*(H) set to 1.5*U*(C).

The amino H-atom was located in a difference Fourier map, and was refined with a distance restraint of N–H 0.84±0.01 Å; its temperature factor was refined.

### Crystal data

**Table:**

C_6_H_10_N_6_O_4_	*F*(000) = 480
*M**_r_* = 230.20	*D*_x_ = 1.437 Mg m^−^^3^
Monoclinic, *P*2_1_/*n*	Mo *K*α radiation, λ = 0.71073 Å
Hall symbol: -P 2yn	Cell parameters from 2279 reflections
*a* = 6.2509 (5) Å	θ = 2.2–26.2°
*b* = 9.1327 (8) Å	µ = 0.12 mm^−^^1^
*c* = 18.6566 (16) Å	*T* = 293 K
β = 92.134 (2)°	Prism, yellow
*V* = 1064.32 (16) Å^3^	0.32 × 0.22 × 0.18 mm
*Z* = 4	

### Data collection

**Table:**

Bruker SMART APEX diffractometer	1711 reflections with *I* > 2σ(*I*)
Radiation source: fine-focus sealed tube	*R*_int_ = 0.023
Graphite monochromator	θ_max_ = 27.5°, θ_min_ = 2.2°
ω scans	*h* = −8→7
6176 measured reflections	*k* = −11→11
2402 independent reflections	*l* = −14→24

### Refinement

**Table:**

Refinement on *F*^2^	Primary atom site location: structure-invariant direct methods
Least-squares matrix: full	Secondary atom site location: difference Fourier map
*R*[*F*^2^ > 2σ(*F*^2^)] = 0.050	Hydrogen site location: inferred from neighbouring sites
*wR*(*F*^2^) = 0.166	H atoms treated by a mixture of independent and constrained refinement
*S* = 1.05	*w* = 1/[σ^2^(*F*_o_^2^) + (0.0903*P*)^2^ + 0.1496*P*] where *P* = (*F*_o_^2^ + 2*F*_c_^2^)/3
2402 reflections	(Δ/σ)_max_ = 0.001
149 parameters	Δρ_max_ = 0.21 e Å^−^^3^
1 restraint	Δρ_min_ = −0.21 e Å^−^^3^

### Fractional atomic coordinates and isotropic or equivalent isotropic displacement parameters (Å^2^)

**Table:**

	*x*	*y*	*z*	*U*_iso_*/*U*_eq_	
O1	0.0322 (3)	0.3824 (2)	0.18681 (9)	0.0936 (6)	
O2	0.2815 (3)	0.3201 (2)	0.38149 (7)	0.0895 (5)	
O4	0.8233 (3)	0.0997 (2)	0.34861 (9)	0.0993 (6)	
O5	0.9040 (3)	0.04877 (19)	0.24053 (10)	0.0906 (5)	
N1	0.3219 (2)	0.25696 (16)	0.15264 (7)	0.0506 (4)	
N2	0.2040 (2)	0.31596 (16)	0.19716 (7)	0.0541 (4)	
N3	0.1665 (3)	0.3517 (2)	0.32062 (9)	0.0761 (5)	
N4	0.4710 (3)	0.2504 (2)	0.36761 (8)	0.0782 (5)	
N5	0.6222 (2)	0.17780 (19)	0.25756 (8)	0.0630 (4)	
N6	0.7946 (3)	0.10260 (19)	0.28501 (10)	0.0678 (5)	
C1	0.4246 (5)	0.1849 (4)	0.03862 (11)	0.1183 (12)	
H1A	0.4277	0.0852	0.0549	0.177*	
H1B	0.5609	0.2299	0.0493	0.177*	
H1C	0.3948	0.1871	−0.0122	0.177*	
C2	0.0397 (4)	0.1911 (4)	0.06269 (13)	0.1051 (9)	
H2A	−0.0692	0.2447	0.0864	0.158*	
H2B	0.0471	0.0932	0.0813	0.158*	
H2C	0.0056	0.1880	0.0121	0.158*	
C3	0.2466 (4)	0.4235 (3)	0.05127 (11)	0.0851 (7)	
H3A	0.1365	0.4744	0.0758	0.128*	
H3B	0.2158	0.4270	0.0005	0.128*	
H3C	0.3822	0.4694	0.0619	0.128*	
C4	0.2546 (3)	0.2664 (2)	0.07571 (9)	0.0600 (5)	
C5	0.2791 (3)	0.30095 (19)	0.27008 (9)	0.0525 (4)	
C6	0.4692 (3)	0.23759 (19)	0.29834 (9)	0.0536 (4)	
H1	0.602 (3)	0.170 (2)	0.2114 (5)	0.056 (5)*	

### Atomic displacement parameters (Å^2^)

**Table:**

	*U*^11^	*U*^22^	*U*^33^	*U*^12^	*U*^13^	*U*^23^
O1	0.0717 (9)	0.1239 (14)	0.0855 (11)	0.0512 (9)	0.0053 (8)	0.0056 (9)
O2	0.1138 (13)	0.1042 (12)	0.0515 (8)	0.0024 (10)	0.0179 (8)	−0.0145 (8)
O4	0.0878 (11)	0.1234 (14)	0.0836 (11)	0.0008 (10)	−0.0391 (9)	0.0258 (10)
O5	0.0688 (10)	0.0861 (11)	0.1168 (13)	0.0227 (8)	0.0031 (10)	0.0077 (10)
N1	0.0488 (7)	0.0592 (8)	0.0438 (7)	0.0092 (6)	−0.0008 (6)	−0.0007 (6)
N2	0.0491 (8)	0.0583 (8)	0.0549 (8)	0.0101 (6)	0.0022 (6)	0.0009 (6)
N3	0.0848 (12)	0.0828 (12)	0.0617 (10)	0.0064 (10)	0.0172 (9)	−0.0119 (9)
N4	0.1005 (14)	0.0881 (13)	0.0456 (8)	−0.0036 (11)	−0.0042 (9)	−0.0002 (8)
N5	0.0591 (9)	0.0774 (11)	0.0520 (8)	0.0157 (8)	−0.0053 (7)	0.0085 (8)
N6	0.0552 (9)	0.0646 (10)	0.0825 (12)	−0.0009 (8)	−0.0130 (8)	0.0146 (9)
C1	0.136 (2)	0.167 (3)	0.0522 (12)	0.068 (2)	0.0072 (14)	−0.0082 (15)
C2	0.110 (2)	0.131 (2)	0.0727 (14)	−0.0425 (18)	−0.0282 (14)	0.0022 (15)
C3	0.1064 (18)	0.0854 (15)	0.0623 (12)	−0.0030 (13)	−0.0119 (12)	0.0188 (11)
C4	0.0629 (11)	0.0721 (12)	0.0443 (9)	0.0054 (9)	−0.0068 (7)	0.0025 (8)
C5	0.0587 (10)	0.0507 (9)	0.0485 (9)	−0.0033 (7)	0.0088 (7)	−0.0030 (7)
C6	0.0646 (10)	0.0516 (9)	0.0442 (8)	−0.0055 (8)	−0.0017 (7)	0.0036 (7)

### Geometric parameters (Å, º)

**Table:**

O1—N2	1.2417 (19)	C1—C4	1.489 (3)
O2—N3	1.352 (2)	C1—H1A	0.9600
O2—N4	1.378 (3)	C1—H1B	0.9600
O4—N6	1.194 (2)	C1—H1C	0.9600
O5—N6	1.200 (2)	C2—C4	1.520 (3)
N1—N2	1.2527 (18)	C2—H2A	0.9600
N1—C4	1.483 (2)	C2—H2B	0.9600
N2—C5	1.429 (2)	C2—H2C	0.9600
N3—C5	1.284 (2)	C3—C4	1.506 (3)
N4—C6	1.297 (2)	C3—H3A	0.9600
N5—C6	1.358 (2)	C3—H3B	0.9600
N5—N6	1.362 (2)	C3—H3C	0.9600
N5—H1	0.869 (9)	C5—C6	1.407 (2)
			
N3—O2—N4	112.00 (13)	C4—C2—H2C	109.5
N2—N1—C4	117.65 (13)	H2A—C2—H2C	109.5
O1—N2—N1	129.32 (15)	H2B—C2—H2C	109.5
O1—N2—C5	116.51 (15)	C4—C3—H3A	109.5
N1—N2—C5	114.15 (13)	C4—C3—H3B	109.5
C5—N3—O2	104.54 (17)	H3A—C3—H3B	109.5
C6—N4—O2	104.67 (17)	C4—C3—H3C	109.5
C6—N5—N6	123.77 (16)	H3A—C3—H3C	109.5
C6—N5—H1	120.6 (12)	H3B—C3—H3C	109.5
N6—N5—H1	114.5 (13)	C1—C4—N1	103.85 (15)
O4—N6—O5	127.57 (19)	C1—C4—C3	110.6 (2)
O4—N6—N5	118.2 (2)	N1—C4—C3	110.69 (16)
O5—N6—N5	114.21 (17)	C1—C4—C2	110.0 (2)
C4—C1—H1A	109.5	N1—C4—C2	110.20 (16)
C4—C1—H1B	109.5	C3—C4—C2	111.28 (19)
H1A—C1—H1B	109.5	N3—C5—C6	110.60 (16)
C4—C1—H1C	109.5	N3—C5—N2	119.65 (17)
H1A—C1—H1C	109.5	C6—C5—N2	129.75 (15)
H1B—C1—H1C	109.5	N4—C6—N5	127.89 (18)
C4—C2—H2A	109.5	N4—C6—C5	108.18 (17)
C4—C2—H2B	109.5	N5—C6—C5	123.90 (15)
H2A—C2—H2B	109.5		
			
C4—N1—N2—O1	0.8 (3)	N1—N2—C5—N3	−177.04 (17)
C4—N1—N2—C5	179.52 (14)	O1—N2—C5—C6	−177.20 (18)
N4—O2—N3—C5	1.0 (2)	N1—N2—C5—C6	3.9 (3)
N3—O2—N4—C6	−1.0 (2)	O2—N4—C6—N5	179.06 (18)
C6—N5—N6—O4	−6.7 (3)	O2—N4—C6—C5	0.6 (2)
C6—N5—N6—O5	174.83 (19)	N6—N5—C6—N4	9.1 (3)
N2—N1—C4—C1	−179.1 (2)	N6—N5—C6—C5	−172.60 (17)
N2—N1—C4—C3	62.2 (2)	N3—C5—C6—N4	0.0 (2)
N2—N1—C4—C2	−61.4 (2)	N2—C5—C6—N4	179.17 (17)
O2—N3—C5—C6	−0.7 (2)	N3—C5—C6—N5	−178.51 (18)
O2—N3—C5—N2	−179.88 (15)	N2—C5—C6—N5	0.6 (3)
O1—N2—C5—N3	1.8 (3)		

### Hydrogen-bond geometry (Å, º)

**Table:**

*D*—H···*A*	*D*—H	H···*A*	*D*···*A*	*D*—H···*A*
N5—H1···N1	0.87 (1)	2.18 (2)	2.758 (2)	124 (2)
